# Generic and Model-Based Calibration Method for Spatial Frequency Domain Imaging with Parameterized Frequency and Intensity Correction

**DOI:** 10.3390/s23187888

**Published:** 2023-09-14

**Authors:** Stefan A. Lohner, Steffen Nothelfer, Alwin Kienle

**Affiliations:** Institut für Lasertechnologien in der Medizin und Meßtechnik an der Universität Ulm, Helmholtzstr. 12, D-89081 Ulm, Germany; steffen.nothelfer@ilm-ulm.de (S.N.); alwin.kienle@ilm-ulm.de (A.K.)

**Keywords:** spatial frequency domain imaging, scattering, absorption, camera calibration, pinhole camera model

## Abstract

Spatial frequency domain imaging (SFDI) is well established in biology and medicine for non-contact, wide-field imaging of optical properties and 3D topography. Especially for turbid media with displaced, tilted or irregularly shaped surfaces, the reliable quantitative measurement of diffuse reflectance requires efficient calibration and correction methods. In this work, we present the implementation of a generic and hardware independent calibration routine for SFDI setups based on the so-called pinhole camera model for both projection and detection. Using a two-step geometric and intensity calibration, we obtain an imaging model that efficiently and accurately determines 3D topography and diffuse reflectance for subsequently measured samples, taking into account their relative distance and orientation to the camera and projector, as well as the distortions of the optical system. Derived correction procedures for position- and orientation-dependent changes in spatial frequency and intensity allow the determination of the effective scattering coefficient $\mu_{s}^{\prime }$ and the absorption coefficient $\mu_{a}$ when measuring a spherical optical phantom at three different measurement positions and at nine wavelengths with an average error of 5% and 12%, respectively. Model-based calibration allows the characterization of the imaging properties of the entire SFDI system without prior knowledge, enabling the future development of a digital twin for synthetic data generation or more robust evaluation methods.

## 1. Introduction

Spatial frequency domain imaging (SFDI) is a non-contact and fast measurement method to determine both the 3D shape and optical properties of scattering samples. A major advantage is the high lateral resolution that can be achieved to quantitatively map, for example, the effective volume scattering coefficient $\mu_{s}^{\prime }$, the absorption coefficient $\mu_{a}$, the phase function parameter γ or the surface scattering parameter $r_{s}$ [1,2]. The technology has considerable applications in biology and medicine, where the separation of scattering and absorption can provide valuable information for imaging different tissue types [3,4,5]. The measurement principle is based on the projection of periodic patterns onto a sample, usually sinusoidally modulated along one spatial direction, and the detection of the diffusely scattered light with a camera. A frequently used method is phase shifting, i.e., subsequently recording a sequence of phase-shifted sinusoidal patterns to calculate the amplitude modulation (AC), phase and offset (DC) using a single-pixel demodulation algorithm [6]. Other methods aim to reduce the number of projections to ideally only a single snapshot to enable real-time measurements based on multi-pixel demodulation [7,8]. In both cases, the individual modulation transfer function (MTF) describing the response of the optical system at different spatial frequencies must be taken into account. The system’s MTF can be determined, for example, by measuring a reference object with known optical properties. In subsequent measurements, the MTF of the system can then be separated from the amplitude modulation emanating from the sample, i.e., the sample’s MTF, also called the diffuse reflection [9]. Based on this characteristic quantity, the optical properties of the sample, e.g., $\mu_{s}^{\prime }$ and $\mu_{a}$, can be determined by the regression of a suitable light propagation model such as the diffusion equation or an analytical solution of the radiative transfer equation [10,11]. In addition, approaches based on machine learning and deep learning are gaining importance [12,13,14]. However, regardless of the evaluation method, the determination of diffuse reflectance is limited in accuracy and susceptible to various sources of error, especially when measuring samples with irregularly shaped or inclined surfaces and varying working distances [15]. This raises the question of how to achieve robust and repeatable system calibration and data acquisition for the typical measurement conditions encountered in the study of biological tissue.

In the simplified case of a flat sample, the diffuse reflectance can be corrected by measuring a reference sample with known optical properties at exactly the same position [10]. For more complex sample surfaces, various profile corrections have been reported based on phase profilometry [16,17,18,19]. In addition to triangulation-based approaches, which require knowledge of the system parameters, e.g., the relative position between the projector and camera, multi-height calibration, i.e., measuring a reference sample at known relative heights, is well established [20,21]. In both cases, a linear relationship between phase and height change relative to a defined reference plane is assumed. For additional angular corrections, the positions and optical axes of the camera and projector relative to the sample must be known or sufficiently accurately estimated. Both the calibration effort and the required prior knowledge can be reduced by an improved modeling of the system. In this context, the pinhole camera model introduced by Zhang [22] is noteworthy, originally developed for camera calibration and later extended for projector-camera systems focusing on improved 3D data determination [23,24,25]. The advantages of an easy-to-use calibration routine combined with the comprehensive characterization of imaging properties motivated the application and extension of the pinhole model in the field of structured illumination.

In this paper, we present a two-step calibration routine for SFDI systems: geometric calibration involves measuring a calibration target with a circular grid at different positions to determine the imaging characteristics and distortion of the projector and camera using the pinhole camera model. After transformation to a uniform coordinate system, the geometry of the projector-camera system can be described consistently, which is used to calculate a 3D map of the sample surface using a nonlinear phase distance model. Thus, for each 3D point, the relative distance to the camera and the projector as well as the projection and acquisition angle can be specified in cylindrical coordinates. Knowledge of the distortion parameters allows for their correction after image acquisition and the calculation of pre-distorted phase images, which produce sinusoidal patterns of high homogeneity when projected onto a reference plane for a given working distance. If the sample surface deviates from the reference surface, the resulting local change in spatial frequency can be quantified geometrically. The second calibration step uses a calibration sample with known optical properties to characterize the MTF of the system and the heterogeneous intensity distribution within the calibrated volume. By parameterization and interpolation within the entire calibrated volume, the model provides the system MTF and reference intensity as a look-up table for subsequent measurements. Optical phantoms were measured for validation, showing how the information from both calibration steps can be efficiently used for post-processing the data without the need for further measurements. In particular, $\mu_{s}^{\prime }$ and $\mu_{a}$ could be determined for a spherical phantom in three different positions with an average deviation of 5% and 12%, respectively, at nine different wavelengths.

The aim of this work was to develop a generic and hardware-independent calibration routine for SFDI setups based on the so-called pinhole camera model for projection and detection. Through a two-step geometric and intensity calibration, we obtain an imaging model that can be used to accurately determine the diffuse reflectance of turbid media with displaced or irregular surfaces. In particular, we present correction methods to account for position- and orientation-dependent changes in the spatial frequency and intensity, which are validated by measuring $\mu_{s}^{\prime }$ and $\mu_{a}$ of a spherical optical phantom at different positions and wavelengths.

## 2. Materials and Methods

### 2.1. Multispectral SFDI Setup

**Figure 1:**
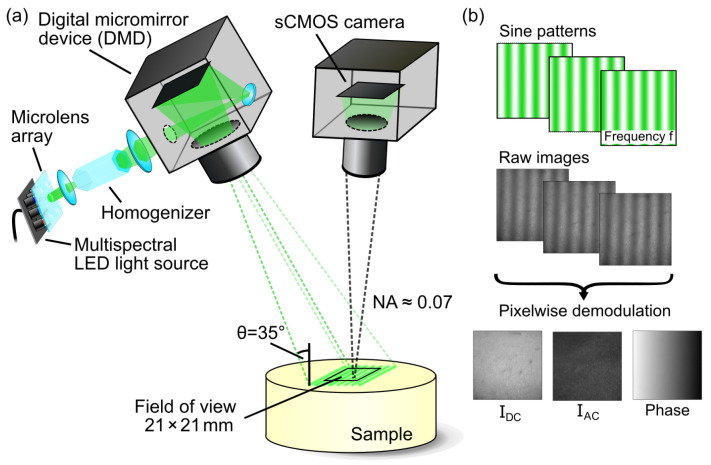
Schematic drawing of (**a**) the multispectral spatial frequency domain imaging (SFDI) setup consisting of a projection unit with a digital micromirror device (DMD), a tunable LED lightsource and a sCMOS camera to detect the diffuse reflect light. (**b**) Pixel-wise demodulation for spatial frequency f yields the offset $I_{DC}$, the modulation amplitude $I_{AC}$ and the unwrapped phase.

Calibration and subsequent measurements were performed using a multispectral SFDI system schematically shown in Figure 1a. The projection unit consists of a digital micromirror device (DLP LightCrafter 6500, Texas Instruments, Dallas, TX, USA) in combination with a self-configured LED light source that provides subsequent illumination based on nine switchable LEDs (XLamp XP-E and XQ-E series, CreeLED, Durham, NC, USA and LUXEON SunPlus series, Lumileds, San Jose, CA, USA) with peak emissions between 447 nm and 945 nm and a narrow bandwidth of about 10 nm each. The light emitted by the LEDs is collimated by a custom microlens array, which, in combination with a light guide homogenization rod, provides virtually homogeneous illumination. The DLP projects sinusoidal intensity patterns at an oblique angle of θ=35° onto the sample and the diffusely reflected light from a 21 mm × 21 mm area is detected by a vertically mounted, cooled sCMOS camera (Zyla 4.2 sCMOS, Andor Technology, Belfast, UK) with a numerical aperture of about 0.07. For the measurement, different spatial frequencies between $0\;{\mathrm{mm}}^{-1}$ and $1\;{\mathrm{mm}}^{-1}$ are recorded with at least three phase shifts of 0, 2π/3 and 4π/3, respectively. The exposure time is about 20 ms per image, resulting in a total measurement time of about 330 ms for five spatial frequencies with a complete sequence of 16 patterns (15 phase patterns + 1 dark pattern) per wavelength. Thus, the multispectral data acquisition takes less than 3 s in total. As shown in Figure 1b, the pixel-wise demodulation provides the offset $I_{DC}$, the modulation amplitude $I_{AC}$ and the phase. A detailed description of the additional phase unwrapping used to avoid phase jumps can be found in Geiger et al. [26].

### 2.2. Calibration Model

The basis for calibration is an appropriate model that describes the imaging process, in the most general case as a mapping of a ray from 3D space to a 2D pixel. Depending on the imaging system, there are both parameterized and non-parameterized models, e.g., for pinhole cameras, stereo cameras, fisheye cameras or catadioptric cameras, as shown in detail by Ramalingam [27] and Grossberg [28]. SFDI systems with commercial camera objectives of a low numerical aperture (NA), i.e., high f-number, can usually be approximated by a pinhole camera, for which Zhang [22] has presented a parameterized calibration model taking distortion into account. This so-called pinhole camera model assumes collinear mapping of object points along straight lines with a common intersection point in the projection center (pinhole) onto an image plane [22]. Mathematically, this corresponds to the transformation of a 3D point ${\vec{P}}_{glob}$ to its 2D projection $\vec{p}=\left(u,v\right)$ on the camera chip given by

(1) $$s\;\vec{p}=A\left[R|t\right]\;{\vec{P}}_{glob},$$

with the scale factor *s*, the camera intrinsic matrix *A* and the extrinsic rotation-translation matrix $\left[R|t\right]$. The extrinsic parameters transform the global coordinates of an object by means of translation *t* and rotation *R* into the local camera coordinate system. Especially for systems consisting of several cameras or projectors, this allows the corresponding coordinates of the subsystems to be correlated. The camera intrinsic matrix

(2) $$A=\left[\begin{array}{ccc} f_{x} & 0 & c_{x}\\ 0 & f_{y} & c_{y}\\ 0 & 0 & 1 \end{array}\right]$$

contains the focal lengths $f_{x}$ and $f_{y}$ and the principal points ($c_{x}$, $c_{y}$), which together with the scale factor are called intrinsic parameters. In addition, the model can be extended to take into account radial distortions that occur in real systems approximated by

(3) $$\begin{array}{ccc} \left[\begin{array}{c} \delta u_{i}^{\left(r\right)}\\ \delta v_{i}^{\left(r\right)} \end{array}\right] & = & \left[\begin{array}{c} u_{i}\left(k_{1}r_{i}^{2}+k_{2}r_{i}^{4}+\cdots \right)\\ v_{i}\left(k_{1}r_{i}^{2}+k_{2}r_{i}^{4}+\cdots \right) \end{array}\right] \end{array}$$

with the radial distortion coefficients $k_{1},k_{2},\cdots k_{n}$ and $r_{i}=\sqrt{u_{i}^{2}+v_{i}^{2}}$, as well as lateral distortions approximated by

(4) $$\begin{array}{ccc} \left[\begin{array}{c} \delta u_{i}^{\left(t\right)}\\ \delta v_{i}^{\left(t\right)} \end{array}\right] & = & \left[\begin{array}{c} 2p_{1}u_{i}v_{i}+p_{2}\left(r_{i}^{2}+2u_{i}^{2}\right)\\ p_{1}\left(r_{i}^{2}+2v_{i}^{2}\right)+2p_{2}u_{i}v_{i} \end{array}\right] \end{array}$$

with the lateral distortion coefficients $p_{1}$ and $p_{2}$ [29].

**Figure 2:**
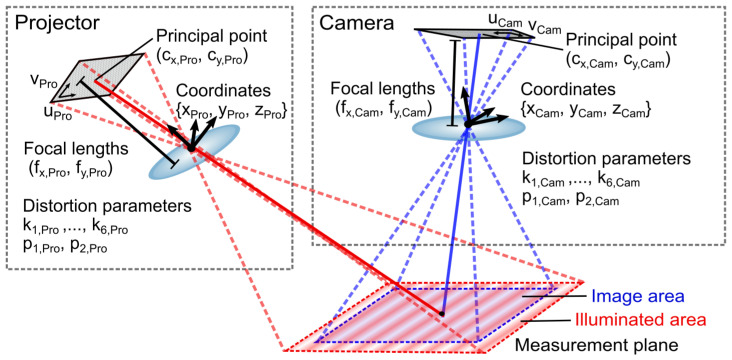
Schematic illustration of the pinhole camera model for a projector–camera system with intrinsic and extrinsic parameters.

Figure 2 shows a schematic illustration of the pinhole camera model for a camera–projector system typically used for SFDI. In this case, both the camera and the projector are described individually by the pinhole camera model with its own set of parameters and coordinates.

There are several implementations of this parameterized pinhole camera model for general calibration purposes, such as the Python module Camera Calibration and 3D Reconstruction from the open source computer vision library (openCV) [30], which was used in this work. For Matlab, an implementation with comparable functionality has recently become available as part of the Camera Calibration Toolbox [31].

### 2.3. Calibration Routine

The calibration of the SFDI system is divided into two parts: the first calibration routine uses a circular grid to characterize the imaging properties of the camera and projector based on the pinhole camera model (referred to as geometric calibration), while afterwards, a calibration standard with known reflectance properties is used for intensity calibration.

#### 2.3.1. Geometric Calibration

The aim of geometric calibration is to determine the extrinsic and intrinsic parameters using the pinhole camera model for both the camera and the projection system. If we first restrict ourselves to camera calibration, according to Zhang [22], it is sufficient to take snapshots of a calibration target in at least three different orientations and distances. The calibration target is essentially a two-dimensional marker structure consisting of regularly and grid-like arranged circles, points or a checkerboard. The two main axes of the grid span a local coordinate system in which each marker point can be uniquely assigned a 2D coordinate (in units of the grid constant *a*), which will be referred to as object points in the following. After capturing the calibration target in different positions, an image processing algorithm recognizes the individual marker structures in each of the resulting camera images. Their pixel coordinates are referred to as image points in the following and, together with the corresponding object points, serve as input parameters for the actual modeling. Estimating the pose of the camera based on a set of 2D points and their corresponding 3D points is also known as the perspective-n-point problem [32]. The solution approach followed in this study, called P3P [33], uses a Levenberg–Marquardt optimization algorithm to determine the rotation, translation and intrinsic parameters according to Equation (1), which minimizes the reprojection error of the 3D–2D point correspondences for all acquired positions of the calibration target. Accordingly, the camera calibration is valid only within the volume originally sampled by the calibration target, hereafter referred to as the calibrated volume. The extent of the calibrated volume is hardware limited, laterally by the field of view and in the z-direction by the depth of field of the camera.

The procedure described for a single camera can be extended to the calibration of a projector using the same pinhole camera model, but with reversed conceptualization. The main difference is the determination of the image points, which are not directly accessible with the projector in contrast to the camera. Instead, phase images are projected onto the calibration target, allowing the pixel rows and columns of the projector chip to be encoded onto the measurement plane. By evaluating the unique phase at each marker structure, the required correspondence can thus be found indirectly. In this work, the phase is determined using the phase shift method, which is based on the demodulation of at least three sequentially recorded sinusoidal patterns, each shifted by 2π/3. The phase patterns are adapted to the resolution of the DMD chip $\left(N_{Col},N_{Row}\right)$ and are projected both along the direction of the pixel columns and rows, while the pixel-based spatial frequencies $f_{Col}$ and $f_{Row}$ have to be specified in each case. Under this condition, the measured phase $\phi_{u}$ and $\phi_{v}$ for patterns along rows and columns, respectively, can be uniquely associated with a particular pixel coordinate $\left(u_{DLP},\;v_{DLP}\right)$ on the DMD chip, according to

(5) $$\begin{array}{cc} u_{DLP} & =\frac{\phi_{u}}{f_{Col}\cdot 4\pi /N_{Col}}, \end{array}$$

(6) $$\begin{array}{cc} v_{DLP} & =\frac{\phi_{v}}{f_{Row}\cdot 4\pi /N_{Row}}. \end{array}$$

Once the two phase values and thus the corresponding image point for the projector calibration are found for each marker point of the calibration target, the subsequent modeling based on the pinhole camera model follows the approach already described for the camera.

**Figure 3:**
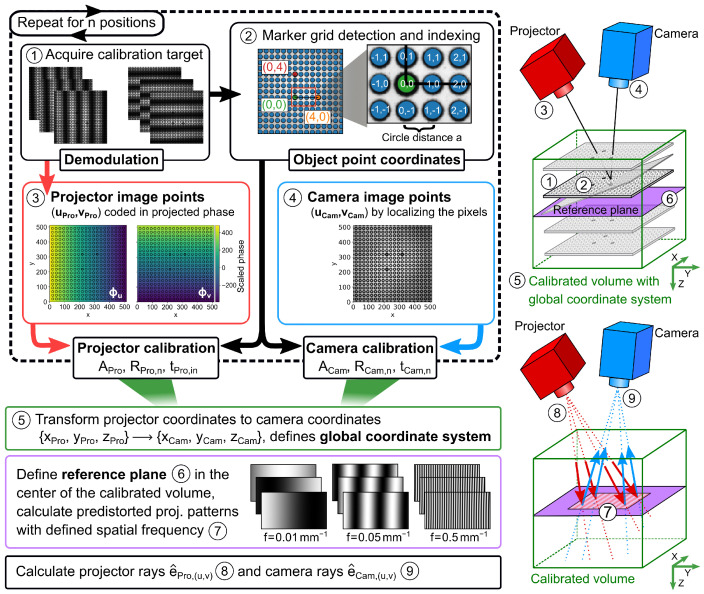
Schematic illustration with an overview of the processing steps for the geometric calibration.

To better illustrate the combined calibration of the camera and projector, an overview of the processing steps for the geometric calibration is shown in Figure 3. In step ➀, the calibration target is positioned in the measurement field and three phase patterns are recorded along the columns and rows of the projector. After demodulation, in step ➁, we use the DC images (corresponding to images under homogeneous illumination) to detect the marker circles using the computer vision functionality of the openCV. Three points with a thicker outline, thus distinguishable from the other points, define the origin and the main axes of the object points with known distance *a*. In step ➂, the pixel positions in the camera image, i.e., the camera image points, are determined for each marker. In step ➃, $\phi_{v}$ and $\phi_{u}$ are determined for each marker circle from the demodulated and unwrapped phase images. Equation (5) yields the projector image points. Steps ➀ to ➃ are then repeated for *n* different positions, but at least three times. The camera pinhole model is then determined separately for the camera and projector image points of all *n* positions with their respective object points, resulting in their intrinsic and extrinsic parameters. Although this technically completes the geometric calibration, there are some useful post-processing steps. In step ➄, we define, without a loss of generality, a global coordinate system by transforming the projector coordinates into the camera coordinate system. The transformation matrix is obtained by transforming $\left[R_{\mathrm{Pro},n},t_{\mathrm{Pro},n}\right]$ to $\left[R_{\mathrm{Cam},n},t_{\mathrm{Cam},n}\right]$. In step ➅, we define a reference plane in the center of the previously calibrated volume, aligned parallel to the camera chip. Using the imaging model of the projector and the distortion parameters, step ➆ computes a series of predistorted phase patterns that, when projected onto the reference plane, yield equidistant sinusoidal fringes with selectable spatial frequencies in units of ${\mathrm{mm}}^{-1}$, ranging from $0\;{\mathrm{mm}}^{-1}$ to $1\;{\mathrm{mm}}^{-1}$. Finally, in steps ➇ and ➈, a normalized direction vector ${\hat{e}}_{Pro,(u,v)}$, ${\hat{e}}_{Cam,(u,v)}$ according to the imaging model is assigned to each pixel of the projector and camera chip, respectively, which will be referred to as projector and camera rays in the following.

#### 2.3.2. Parametrized 3D Point Estimation

Geometric calibration allows a 3D point cloud to be determined by triangulation using the phase information. This means that for a given camera pixel, the measured global phase is directly linked to a unique 3D coordinate. Instead of a strictly geometric determination, in this work, we applied a parameterized approach using the presented pinhole camera model, as reported by Lu [34]. For this purpose, each camera ray defines a vector within the calibrated volume, which is sampled with a certain number of 3D points with known distances $l_{\left(u,v\right)}$ from the camera center. Using the inverted imaging model, each of these 3D points can be associated with the corresponding 2D image point of the projector (i.e., DMD pixel) and thus, with the corresponding global phase $\varphi_{\mathrm{glob},(u,v)}$. An efficient mapping between the scalars $l_{\left(u,v\right)}$ and $\varphi_{\mathrm{glob},(u,v)}$ is obtained by the regression of a cubic polynomial for each camera ray according to

(7) $$\begin{array}{c} l_{\left(u,v\right)}=P\left(\varphi_{\mathrm{glob},(u,v)}\right)=\sum_{k=0}^{n=3}a_{k,(u,v)}\cdot \varphi_{\mathrm{glob},(u,v)}. \end{array}$$

Once the coefficients $a_{k}$ are determined, the distances $l_{\left(u,v\right)}$ for all camera pixels of a measured phase image can be computed efficiently and very quickly in the following, using a hyperbolic solution [35], which we call the phase-distance conversion in the following. Additional multiplication with the camera rays ${\hat{e}}_{Cam,(u,v)}$, i.e., the direction vectors, yields the corresponding 3D coordinate:

(8) $$\begin{array}{c} {\vec{P}}_{glob,(u,v)}={\hat{e}}_{\mathrm{cam},(u,v)}\cdot l_{\left(u,v\right)}={\left(x,y,z\right)}_{\left(u,v\right)}. \end{array}$$

#### 2.3.3. Calculating Normals and Angles for Spatial Frequency Correction

**Figure 4:**
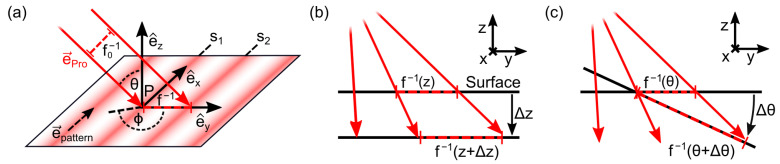
(**a**) Schematic representation of a sinusoidal pattern with spatial frequency $f_{0}$ projected obliquely onto a plane surface in the local coordinate system of a point $\vec{P}$. (**b**) Both a height shift of Δ*z* and (**c**) an inclination of the sample relative to the reference plane by Δθ lead to scaling of the local spatial frequency *f* observed on the sample surface.

For the further post-processing of the point cloud, the open source library Open3D [36] was used to calculate a normal vector ${\vec{n}}_{P}$ for each 3D point ${\vec{P}}_{glob}$ considering its nearest neighbors. The normal vectors ${\vec{n}}_{P}$, together with the corresponding projector rays ${\vec{e}}_{Pro,P}$ and the direction of the fringe maxima ${\vec{e}}_{pattern}$ (i.e., the direction of the constant phase in the reference plane), span a local coordinate system for every 3D point with the direction vectors given by

(9) $$\begin{array}{c} {\vec{e}}_{z,P}={\vec{n}}_{z,P},\;{\vec{e}}_{x,P}={\vec{e}}_{pattern}-{\vec{e}}_{Pro,P}\left(\frac{{\vec{n}}_{P}\cdot {\vec{e}}_{pattern}}{{\vec{n}}_{p}\cdot {\vec{e}}_{Pro,P}}\right)\;\mathrm{and}\;{\vec{e}}_{y,P}={\vec{e}}_{z,p}\times {\vec{e}}_{x,P}, \end{array}$$

as shown schematically in Figure 4a. After nominating the direction vectors via

(10) $$\begin{array}{c} {\hat{e}}_{x,P}=\frac{{\vec{e}}_{x,P}}{\left|{\vec{e}}_{x,P}\right|},\;{\hat{e}}_{y,P}=\frac{{\vec{e}}_{y,P}}{\left|{\vec{e}}_{y,P}\right|},\;{\hat{e}}_{z,P}=\frac{{\vec{e}}_{z,P}}{\left|{\vec{e}}_{z,P}\right|}, \end{array}$$

we can calculate the rotation matrix

(11) $$\begin{array}{c} R_{g,P}={\left({\hat{e}}_{x,P},{\hat{e}}_{y,P},{\hat{e}}_{z,P}\right)}^{-1}, \end{array}$$

which transforms the camera and projections rays from global to local coordinates according to

(12) $$\begin{array}{cc} {\hat{e^{\prime }}}_{Pro,P} & =R_{g,P}\cdot {\hat{e}}_{Pro,P}, \end{array}$$

(13) $$\begin{array}{cc} {\hat{e^{\prime }}}_{Cam,P} & =R_{g,P}\cdot {\hat{e}}_{Cam,P}. \end{array}$$

After the transformation from cartesian to spherical coordinates according to

(14) $$\begin{array}{c} \theta =\arccos\left(\frac{{\hat{e}}_{z,P}^{\prime }\cdot {\vec{n}}_{P}}{|{\hat{e}}_{z,P}^{\prime }\cdot {\vec{n}}_{P}|}\right),\;\phi =\arctan\left(\frac{{\hat{e}}_{y,P}^{\prime }\cdot {\vec{n}}_{P}}{|{\hat{e}}_{y,P}^{\prime }\cdot {\vec{n}}_{P}|},\;\frac{{\hat{e}}_{x,P}^{\prime }\cdot {\vec{n}}_{P}}{|{\hat{e}}_{x,P}^{\prime }\cdot {\vec{n}}_{P}|}\right),\; \end{array}$$

we obtain the projection angles $\left(\theta_{Pro},\phi_{Pro}\right)$ and detection angles $\left(\theta_{Cam},\phi_{Cam}\right)$ for each 3D point, respectively.

As shown in Figure 4a, the oblique projection of a fringe pattern with a spatial period $f_{0}^{-1}$ onto the sample surface results in a scaled spatial period $f^{-1}$, i.e., a changed distance between two fringe maxima, denoted by $s_{1}$ and $s_{2}$. The scaling factor $S_{ang,\|}$ can be described geometrically for a tilt parallel to the fringes as

(15) $$\begin{array}{c} S_{ang,\|}=\sqrt{\cos{\left(\theta_{Pro,P}\right)}^{2}+\sin{\left(\phi_{Pro,P}\right)}^{2}\sin{\left(\theta_{Pro,P}\right)}^{2}}, \end{array}$$

and thus

(16) $$\begin{array}{c} f=S_{ang,\|}\cdot f_{0}. \end{array}$$

The scaling factor $S_{ang,⊥}$ for a tilt perpendicular to the fringes is calculated similarly by

(17) $$\begin{array}{c} S_{ang,⊥,P}=\sqrt{\cos{\left(\theta_{Pro,P}\right)}^{2}+\cos{\left(\phi_{Pro,P}\right)}^{2}\sin{\left(\theta_{Pro,P}\right)}^{2}}. \end{array}$$

Knowing the spatial frequency $f_{ref}$ at a point ${\vec{P}}_{ref,(u,v)}$ in the reference plane illuminated at $\phi_{Pro,ref}$ and $\theta_{Pro,ref}$, the spatial frequency $f_{corr}$ at the point ${\vec{P}}_{\left(u,v\right)}$ of a relatively shifted and tilted surface is given as

(18) $$\begin{array}{c} f=f_{ref}\cdot \;S_{z}\;\cdot S_{ang,\|}\left(\phi_{Pro,P},\theta_{Pro,P}\right) \end{array}$$

with the magnification factor

(19) $$\begin{array}{c} S_{z}=\frac{|{\vec{P}}_{ref}|}{|\vec{P}|}. \end{array}$$

#### 2.3.4. Intensity Calibration

When determining the absolute diffuse reflectance, the inhomogeneous intensity distribution and the MTF of the system, which were neglected so far during the geometric calibration, must also be taken into account. For this purpose, the intensity calibration is performed in a second step using a reference standard of known diffuse reflectance $R_{SFD,ref}$ measured at $N_{pos}$ (usually 5 to 6) positions evenly distributed over the calibrated volume. At each position, $N_{f}$ spatial frequencies (usually 8 to 9) are uniformly acquired between $0\;{\mathrm{mm}}^{-1}$ and $1\;{\mathrm{mm}}^{-1}$. After demodulation, as part of the data post-processing, we apply all demonstrated tools of geometric calibration to obtain pixel-wise 3D coordinates, normal vectors, angles and corrected spatial frequencies in addition to the AC and DC images. Since the detected intensity depends not only on the absolute position of the reference target, but also on its relative position to the projector and camera, we use a Lambert correction model:

(20) $$\begin{array}{c} S_{Lambert,(u,v)}\left(\theta_{Cam},\theta_{Pro},\phi_{Pro}\right)=\cos\left(\theta_{Cam}\right)\cdot S_{ang,⊥}\left(\theta_{Pro},\phi_{Pro}\right)\cdot S_{ang,\|}\left(\theta_{Pro},\phi_{Pro}\right), \end{array}$$

assuming Lambertian reflection of the reference target as an approximation and thus

(21) $$\begin{array}{cc} I_{DC,ref,corr,(u,v)} & =I_{DC,ref(u,v,)}\cdot S_{Lambert,(u,v)}, \end{array}$$

(22) $$\begin{array}{cc} I_{AC,ref,corr,(u,v)} & =I_{AC,ref(u,v,)}\cdot S_{Lambert,(u,v)}. \end{array}$$

To achieve an efficient assignment and later on, the retrieval of the reference intensities despite the large parameter set, we describe the corrected intensities pixelwise as a function of both their 3D position (e.g., distance $l_{\left(u,v\right)}$ from the camera) and their corrected spatial frequency *f*. Thus, for each camera pixel (u,v), we obtain $N_{f}\cdot N_{pos}$ grid-like arranged reference intensities, which correspond to a 2D hypersurface given by

(23) $$\begin{array}{c} MTF_{ref,(u,v)}\left(l,f\right)=\left\{\begin{array}{cc} I_{DC,ref,corr,(u,v)}\left(l\right) & f=0\\ I_{AC,ref,corr,(u,v)}\left(l,f\right) & f\neq 0. \end{array}\right. \end{array}$$

With additional 2D interpolation, the intensity reference $MTF_{ref,(u,v)}\left(l,f\right)$ can be approximated within the entire calibrated volume and stored in the form of a look-up table.

### 2.4. Phantom Measurements

After measuring a sample such as an optical phantom, we repeat the evaluation steps shown so far. The geometric calibration provides a 3D model of the sample surface with corrected spatial frequencies $f_{u,v}$ and angles $\left(\theta_{Pro},\phi_{Pro}\right)$ and $\left(\theta_{Cam},\phi_{Cam}\right)$. The diffuse reflectance $R_{SFD}$ is obtained by

(24) $$\begin{array}{c} R_{SFD,(u,v)}\left(f\right)=\left\{\begin{array}{cc} \sum_{i}^{n}\left(\frac{I_{DC,(u,v,)}\left(f_{i}\right)\cdot S_{Lambert,(u,v)}}{MTF_{ref}\left(l,0\right)}\cdot R_{SFD,ref}\left(0\right)\right)/n & f=0\\ \frac{I_{AC,(u,v,)}\left(f_{i}\right)\cdot S_{Lambert,(u,v)}}{MTF_{ref}\left(l,f\right)}\cdot R_{SFD,ref}\left(f_{i}\right) & f\neq 0. \end{array}\right. \end{array}$$

**Figure 5:**
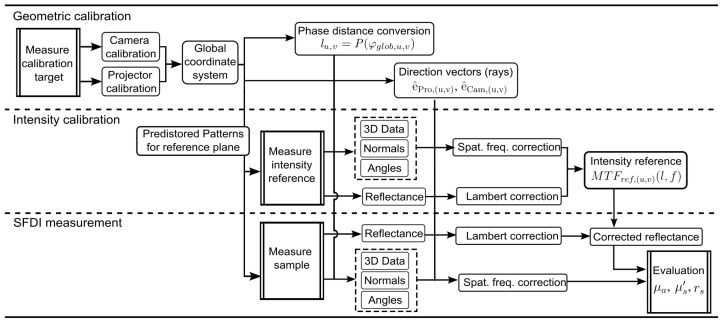
Flowchart showing an overview of the calibration routines and processing steps with spatial frequency and intensity correction applied to an SFDI measurement.

As a summary, both the calibration routines and the subsequent calculation steps for the actual measurement are shown in Figure 5 as a flowchart.

To evaluate the optical properties of the measured sample, we use an analytical solution of the radiative transfer equation based on the $P_{N}$ approximation for semi-infinite media to model light propagation [11,37,38]. The model was applied to the post-processed data using a nonlinear least squares algorithm with a computational accuracy of order N=11, resulting in $\mu_{a}$, $\mu_{s}^{\prime }$ and the surface roughness parameter $r_{s}$ for each pixel. Additional 16×16 binning reduced both evaluation time and data volume.

Optical phantoms made of epoxy resin with additional scattering particles (titanium dioxide) and a mineral absorber (hematite) were used for the validation measurements. The geometry and surface properties of the phantoms can be controlled by molding or mechanical finishing. In the present case, flat cylindrical phantoms and spherical caps of different sizes were used. The reference values of their optical properties were determined with an integrating sphere [39].

## 3. Results and Discussion

### 3.1. Geometric Model of the SFDI Setup in Global Coordinates

**Figure 6:**
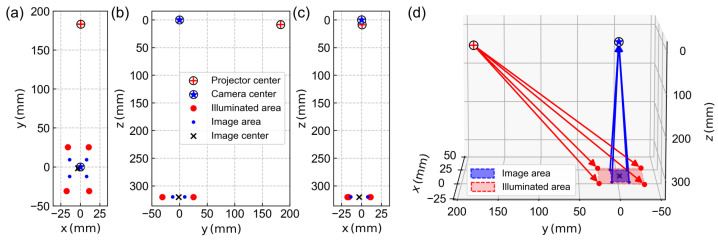
(**a**–**c**) 2D representation of the virtual camera and projector center, the illuminated area and the image area in the global coordinate system as they result from the geometric calibration. (**d**) The corresponding 3D illustration with the outer corner rays of both the camera (blue) and projector (red).

For the geometric calibration of the SFDI device, an aluminum plate with a printed grid of 40×40 circles with a 1 mm diameter and 2 mm spacing was used. The depth of field of the camera limited the volume for calibration to a lateral 21mm×21mm with a height of 25mm centered around the focus position. Since the grid spacing enters Equation (1) via the scaling factor, the extrinsic parameters shown in Figure 6 result directly in millimeter units. The origin of the global coordinate system was chosen at the position of the virtual camera center, the reference plane is at a distance of z=320mm below and the virtual projector center is at (0.61mm,183.0mm,8.6mm). The model predicts a camera field of view in the reference plane (hereafter called the image area) with a lateral extent of 21.6mm× 21.6 mm, while the projector field of view (hereafter called the illuminated area) has a lateral extent of 56.8mm×27.9mm. The camera image center in the reference plane is at (−2.9mm,−1.5mm,320mm), i.e., the optical axis of the camera appears to be aligned almost exactly perpendicular to the reference plane.

Compared to the real SFDI setup, some of these parameters can be directly validated, e.g., the size of the camera field of view in the reference plane can be easily measured and checked with a ruler. Regarding the illumination field, it should be noted that the DMD chip may not be fully illuminated and its margins are usually obscured by parts of the projection optics. The fact that the illumination field is significantly larger than the camera image serves to avoid marginal light propagation effects. The relative position of the camera, projector and reference plane could also be roughly confirmed with a tape measure, although the model parameters do not necessarily reflect the exact setup geometry. In particular, the virtual camera and projector centers are not characteristic or distinctive points of the real optical system. Strictly speaking, their specification is only meaningful if the entire set of model parameters is taken into account.

### 3.2. Phase-Distance Conversion

**Figure 7:**
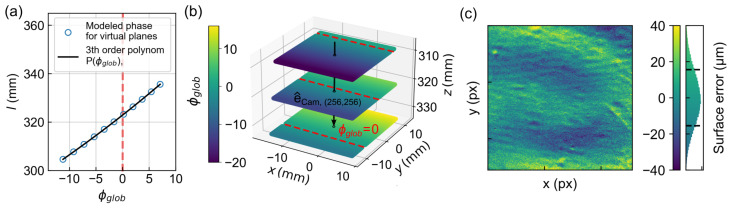
For a single pixel, e.g., in the center of the CCD, $\phi_{glob}$ can be described as a function of the distance $l_{\left(u,v\right)}$ between the sample surface and the camera (blue dots). Fitting a third degree polynomial gives the analytical phase–distance conversion (black line). (**b**) shows three planes with a distance of 10 mm, centered around the reference plane, with color coding showing $\phi_{glob}$. The black arrow corresponds to the profile shown in (**a**) while the red dashed line marks $\phi_{glob}=0$ in each case. (**c**) For validation, we measured the 3D topography of a flat reference target using the presented phase–distance conversion. The mean surface deviation was determined by the regression of a plane as 15μm.

The geometric calibration allows the translation of the measured and unwrapped phase directly into a distance $l_{\left(u,v\right)}$ according to Equation (7). To determine this correspondence, ten virtual planes were defined in the calibrated volume, having been shifted in the z-direction. The projected phase as seen from the camera was then calculated for each plane using the known imaging model, shown exemplarily in Figure 7a for the pixel in the center of the camera. By fitting a third-degree polynomial according to Equation (7), the unique phase–distance relationship can be determined and stored for each pixel. In the subsequent measurement, it is sufficient to specify the camera pixel index, the phase value and the camera ray ${\hat{e}}_{\mathrm{Cam},(u,v)}$ to calculate $l_{\left(u,v\right)}$ or, according to Equation (8), the corresponding 3D coordinate directly. Figure 7b shows three depicted planes in a 3D representation with color-coded $\phi_{glob}$, which can be assigned to a specific column of the DMD chip according to Equation (5). Due to the oblique projection direction, $\phi_{glob}=0$ appears in the camera image at a different position in each plane. The black arrow again represents the camera ray of the center pixel. For validation, a flat reference target was measured in the reference plane and its 3D topography was determined using the phase–distance conversion. Figure 7c shows the surface error of the obtained point cloud determined by the regression of a plane to an average deviation of about 15μm.

### 3.3. Calculating the Projection and Detection Angles

**Figure 8:**
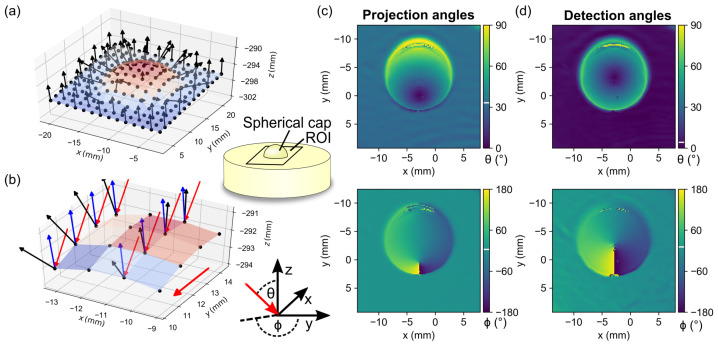
(**a**) 3D topography of an optical phantom with a centered spherical cap showing the normal vectors (black arrows) and color-coded z-coordinate. (**b**) Detailed view with the additional marking of the incident projector direction (red arrows) and the camera detection (blue arrows). (**c**,**d**) show the polar angles $\left(\theta_{Cam},\theta_{Pro}\right)$ and $\left(\phi_{Cam},\phi_{Pro}\right)$, respectively, with the mean angles indicated by a white bar in the colorbar.

To illustrate the determination of the projection and detection angles, a custom-made planar phantom with a central spherical cap with a diameter of approximately 10 mm was measured. Figure 8a shows the 3D point cloud obtained by the phase–distance conversion, with 8×8 pixel binning chosen for clarity. The black arrows illustrate the normal vectors computed for each pixel, while the color map codes are $l_{\left(u,v\right)}$. Figure 8b shows an enlarged section of the point cloud with the additional representation of the camera rays (blue) and projector rays (red) as well as the propagation direction of the fringe pattern (red). Figure 8c,d show the polar angles calculated according to Equation (14) for projection and detection, respectively. The incidence angle of the projection $\phi_{Pro}$ is on average 35°, the detection angle $\phi_{Cam}$ of the camera is about 4° and $\theta_{Cam}$ and $\theta_{Pro}$ are on average 0°.

### 3.4. Look-Up Table for Intensity Reference

**Figure 9:**
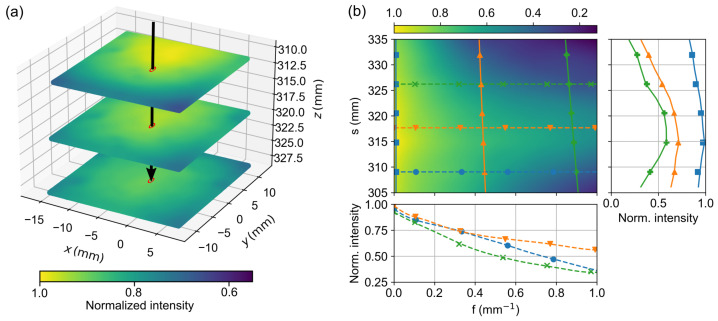
(**a**) Three-dimensional representation of the DC images for three intensity reference measurements, each shifted by Δz=10mm in the z-direction, with an exemplary camera ray marked in the center of the image (black arrow). (**b**) shows for this selected ray the reflectance resulting from the intensity calibration, which is plotted in 2D, color-coded against spatial frequency and distance. The right plot shows an example of the change in reflectance at $f=0\;{\mathrm{mm}}^{-1}$, $f=0.4\;{\mathrm{mm}}^{-1}$, and $f=0.8\;{\mathrm{mm}}^{-1}$ for different distances as solid lines, and the bottom plot shows the MTF in the range between $0\;{\mathrm{mm}}^{-1}$ and $1\;{\mathrm{mm}}^{-1}$ for distances 310 mm, 320 mm and 325 mm as dashed lines.

For the intensity calibration, a white coated, highly scattered aluminum plate with known reflectance properties was used as a reference target for further intensity calibration. It was measured at six different positions in the calibrated volume, each at nine spatial frequencies ranging from $0\;{\mathrm{mm}}^{-1}$ to $1\;{\mathrm{mm}}^{-1}$. Figure 9a shows the corresponding DC images for measurements at three positions with a respective distance of 10 mm. It can be clearly seen that the intensity distribution is not homogeneous and changes laterally as well as for different heights. The black arrow marks the camera ray associated with the pixel at the center of the CCD chip. Figure 9b shows, for this pixel, the corresponding reflectance reference map $MTF_{ref}{\left(l,f\right)}_{\left(u,v\right)}$ obtained by an 2D interpolation of the acquired measurements. It shows the color-coded reflectance for this pixel as a function of distance $l_{\left(u,v\right)}$ from the camera along ${\hat{e}}_{\mathrm{Cam},(u,v)}$ and as a function of the spatial frequency *f*. The outset on the right shows three intensity profiles as a function of $l_{\left(u,v\right)}$ but constant spatial frequencies at $0.01\;{\mathrm{mm}}^{-1}$, $0.43\;{\mathrm{mm}}^{-1}$ and $0.87\;{\mathrm{mm}}^{-1}$, corresponding to the vertical lines in Figure 9a. In all three cases, the intensity decreases at short and long distances due to the limited depth of field of the camera. The intensity maximum at 316 mm indicates the distance of the camera focal plane. The outset below shows the change in intensity as a function of the spatial frequency for three fixed distances at about 309mm, 318mm and 326mm. These curves correspond to the horizontal profiles in Figure 9a. Fringes with a high spatial frequency can obviously only be resolved properly near the focal plane, at distances from approx. ±8mm; the decrease of the MTF indicates blurring.

### 3.5. Determining Multispectral Optical Properties of a Hemispherical Phantom

**Figure 10:**
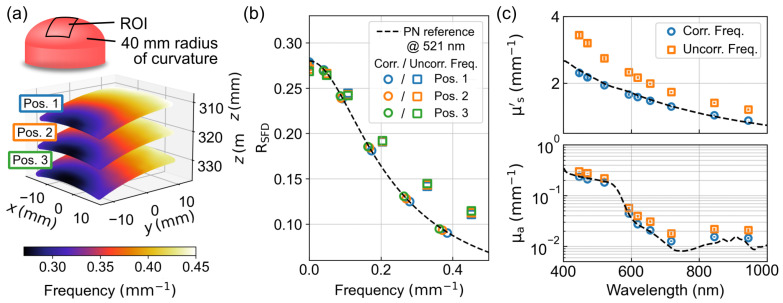
(**a**) Measurement of a hemispherical optical phantom with a radius of curvature of 40 mm in three different z-positions using false colors to display the geometrically corrected spatial frequency for a pattern with $f=0.45\;{\mathrm{mm}}^{-1}$ in the reference plane. (**b**) Averaged reflectances measured at a wavelength of 521 nm after post-processing considering (circles) and neglecting (squares) frequency and intensity correction and compared to a forward calculation. (**c**) Spectrally resolved effective scattering coefficient $\mu_{s}^{\prime }$ and absorption coefficient $\mu_{a}$ determined after post-processing, taking into account (circles) and neglecting (squares) the frequency and intensity correction compared to an integrating sphere measurement.

To evaluate the influence of frequency and intensity corrections on the determination of optical properties, another custom-made hemispherical phantom with a radius of curvature of 40 mm was measured at three different positions in the calibrated volume. The SFD measurement was performed at all nine available wavelengths recording six spatial frequencies between $0.01\;{\mathrm{mm}}^{-1}$ and $0.45\;{\mathrm{mm}}^{-1}$, with respect to the reference plane, and three phase patterns each. Figure 10a shows the three measurement positions where Pos. 2 was approximately at the reference plane and Pos. 1 and Pos. 3 were 10 mm above and below it, respectively. The false colors show the geometrically corrected spatial frequency due to the offset and curvature of the sample for a spatial frequency of $0.45\;{\mathrm{mm}}^{-1}$. A clear gradient between $0.28\;{\mathrm{mm}}^{-1}$ and $0.45\;{\mathrm{mm}}^{-1}$ becomes apparent, which increases with shallower angles between the illumination direction and normal surface. Averaged over the entire field of view, the curvature causes about 15% deviation of the spatial frequency relative to the reference plane, and the height difference between Pos. 1 and Pos. 3 causes an additional variation of about 6%. Figure 10b shows $R_{\mathrm{SFD}}$ at a wavelength of 521nm compared for the post-processing with and without consideration of both the spatial frequency correction and intensity correction. For comparison, the dashed line shows a forward calculation with a semi-infinite solution of the RTE. As a reference, the optical properties were determined by means of an integrating sphere as $\mu_{s}^{\prime }=1.98\;{\mathrm{mm}}^{-1}$ and $\mu_{a}=0.18\;{\mathrm{mm}}^{-1}$, assuming the anisotropy factor g=0.6 and refractive index n=1.52. While the measured $R_{\mathrm{SFD}}$ is shifted to higher spatial frequencies and intensities without corrections, it agrees well with the prediction for all three positions with corrections. The deviations at low spatial frequencies probably result from the deviation from the assumed semi-infinite model. Figure 10c shows the corresponding optical properties for all wavelengths compared with and without correction, with the results of an integrating sphere measurement given as a reference. Without corrections, the deviations for $\mu_{s}^{\prime }$ and $\mu_{a}$ average 35% and 45%, respectively, over all wavelengths, while the deviations with a frequency correction are 5% and 12%, respectively.

## 4. Summary and Outlook

The presented approach of a two-step geometry and intensity calibration allows a comprehensive and coherent description of the entire SFDI system with a large set of internal and external parameters obtained both for the projector and camera. In combination with the 3D topography of the sample, available through the phase information, local changes in spatial frequency or intensity can be determined directly from the displacement or tilt of the sample surface and taken into account in a further analysis. This allows the investigation and further development of correction methods that require virtually no prior knowledge of the setup. The parameterized approach to phase–distance conversion and intensity correction using rays associated with an individual camera and projector pixels provides an efficient and insightful representation of the measurement process and subsequent data processing. A general drawback, however, is the extensive algorithms that must be developed and implemented once for post-processing.

Overall, the results of the calibration, i.e., the characterization of the geometric imaging properties, including realistic distortions and inhomogeneous intensity distributions, can be further used to establish a digital twin of the setup. A future goal is therefore to simulate the entire measurement process with forward calculations using numerical methods such as the Monte Carlo method [40,41]. This would make it possible, for example, to determine the mutual error contribution in the simultaneous determination of the optical properties and the 3D topography of turbid samples or to enable new possibilities for data evaluation. The first progress with a similar approach for the corrected determination of 3D topography of teeth was recently shown by Geiger et al. [26]. In particular, for such complex sample geometries where analytical solutions of the radiative transfer equation do not exist, a regression algorithm to determine the optical properties by solving the inverse problem for virtually any 3D surface would be conceivable.

From an application perspective, the calibration model can provide advantages in various fields, such as medical imaging. After the one-time calibration, which does not require any prior knowledge of the specific hardware used in the application, comprehensive information on the distance and relative orientation of different image areas of the examined specimen is available in addition to the diffuse reflectance. A major advantage is that no additional image acquisition is required for their determination, i.e., the measurement time remains unchanged. In addition to the presented quantitative approach for the determination of scattering and absorption properties, applications in the field of machine learning are becoming more and more important. For example, in the automated classification of tissue types based on reflectance properties, additional knowledge of their relative position and distance to the camera and projector could enable more stable prediction models.

## Data Availability

Data underlying the results presented in this paper are not publicly available at this time but may be obtained from the authors upon request.
